# A Dual Role of P53 in Regulating Colistin-Induced Autophagy in PC-12 Cells

**DOI:** 10.3389/fphar.2017.00768

**Published:** 2017-10-27

**Authors:** Ziyin Lu, Chunli Chen, Zhiyong Wu, Yusong Miao, Ishfaq Muhammad, Liangjun Ding, Erjie Tian, Wanjun Hu, Huilin Ni, Rui Li, Bo Wang, Jichang Li

**Affiliations:** ^1^Department of Veterinary Pharmacology and Toxicology, College of Veterinary Medicine, Northeast Agricultural University, Harbin, China; ^2^Department of Animal Production, College of Life Engineering, Shenyang Institute of Technology, Fushun, China; ^3^Heilongjiang Key Laboratory for Animal Disease Control and Pharmaceutical Development, Harbin, China

**Keywords:** colistin, autophagy, p53, PC-12 cells, apoptosis

## Abstract

This study aimed to investigate the mechanism of p53 in regulating colistin-induced autophagy in PC-12 cells. Importantly, cells were treated with 125 μg/ml colistin for 12 and 24 h after transfection with p53 siRNA or recombinant plasmid. The hallmarks of autophagy and apoptosis were examined by real-time PCR and western blot, fluorescence/immunofluorescence microscopy, and electron microscopy. The results showed that silencing of p53 leads to down-regulation of Atg5 and beclin1 for 12 h while up-regulation at 24 h and up-regulation of p62 noted. The ratio of LC3-II/I and autophagic vacuoles were significantly increased at 24 h, but autophagy flux was blocked. The cleavage of caspase3 and PARP (poly ADP-ribose polymerase) were enhanced, while PC-12-sip53 cells exposed to 3-MA showed down-regulation of apoptosis. By contrast, the expression of autophagy-related genes and protein reduced in p53 overexpressing cells following a time dependent manner. Meanwhile, there was an increase in the expression of activated caspase3 and PARP, condensed and fragmented nuclei were evident. Conclusively, the data supported that silencing of p53 promotes impaired autophagy, which acts as a pro-apoptotic induction factor in PC-12 cells treated with colistin for 24 h, and overexpression of p53 inhibits autophagy and accelerates apoptosis. Hence, it has been suggested that p53 could not act as a neuro-protective target in colistin-induced neurotoxicity.

## Introduction

Colistin (polymyxin E) is the first choice in respect to infections caused by multi-drug-resistant Gram-negative bacteria (MDR-GNB; Bialvaei and Samadi Kafil, 2015), including *Pseudomonas aeruginosa, Acinetobacter baumannii, Klebsiella pneumoniae* (Walkty et al., 2009). It has also been determined to be potentially active against several mycobacterial species, such as *Mycobacterium tuberculosis* (Falagas and Kasiakou, 2005). However, the toxicity (including neurotoxicity and nephrotoxicity) had a direct influence on the effectiveness of colistin in clinic. Its potential neurotoxicity can cause various complications such as perplexity, facial and peripheral paresthesia, muscles weakness, loss of balance, and dyssynergia after intravenous administration of colistin methanesulfonate (CMS, the inactive pro-drug of colistin; Falagas and Kasiakou, 2006; Weinstein et al., 2009; Wahby et al., 2010; Honore et al., 2013). Our colleagues studied that colistin-induced neurotoxicity triggered autophagy and apoptosis in PC-12 cells, and that apoptosis was affected by the control of autophagy (Jiang et al., 2014; Zhang et al., 2015). Importantly, our previous study showed that p53 participates in autophagy and apoptosis in colistin-treated PC-12 cells (Zhang et al., 2016). The PC-12 cell line is derived from pheochromocytoma of the rat adrenal medulla and it is commonly used as a neural differentiation and neurosecretion model (Sasaki et al., 2002; Chen et al., 2013).

Autophagy is a conserved process in which the cytoplasmic contents are first directed toward elimination or turnover in membrane-bound compartments (Pimkina et al., 2009), and then broken down by the lysosomal system. It serves as a protective mechanism against the cellular stress induced by chemotherapy (Ryan, 2011), and also a contributing factor in cell death due to lack of energy. The current evidence also suggested that autophagy indirectly facilitate cell death (Wang et al., 2011). Apoptosis is a morphological and biochemical description of the major mode of a physiological cell death (Kerr et al., 1972); it has been widely appreciated as a key mechanism of regulated death, involved in cell damage/stress and normal development and morphogenesis (Nikoletopoulou et al., 2013). Both apoptosis and autophagy are important in the normal development, physiology and pathology of the cells, and critical in deciding the fate of the cells (Thorburn, 2008). P53 is a tumor suppressor protein, it can trigger cell cycle arrest, allow DNA damage repair and can promote apoptosis when cells are challenged by severe irreparable insults (Maiuri et al., 2010). P53-induced autophagy may either contribute to cell death (Crighton et al., 2007) or constitute a physiological cellular defense response (Amaravadi et al., 2007).

## Materials and methods

### Reagents and antibodies

Colistin sulfate (20,195 U/mg) was purchased from Sigma-Aldrich (lot number 095K1048; St. Louis, MO). Fetal bovine serum (FBS) was obtained from Gibco BRL (Gaithersburg, MD, USA) and Dulbecco's modified Eagle's medium (DMEM), Lipofectamine 2000™, and opti-MEM were obtained from Life Technologies Corporation (Grand Island, NY, USA). 4′,6-diamidino-2-phenylindole (DAPI), 3-MA (autophagy inhibitor) and bafilomycin A1 (BFA) were purchased from Sigma Chemical Co. (St. Louis, Missouri, USA). Primary monoclonal antibodies of anti-beclin1, anti-LC3-II/I (microtubule-associated protein1 light chain 3) and anti-caspase3 were purchased from Cell Signaling Technology (Beverly, MA). Anti-β-action rabbit monoclonal antibody and secondary antibodies (horseradish peroxidase [HRP]-labeled goat anti-rabbit IgG) were obtained from Beijing Zhongshan Golden Bridge Biotechnology Co. Ltd. (Beijing, China).

### Cell lines and culture

Rat PC-12 cells were obtained from the Cell Bank of Type Culture Collection, Shanghai Institute of Cell Biology, Chinese Academy of Sciences. The cells were cultured at 37°C 10% FBS/DMEM in 5% CO_2_ atmosphere in a CO_2_ incubator as described previously (Jiang et al., 2014).

### RNAi and plasmids

Small interfering RNAs targeting p53 were obtained from GenePharma Co. Ltd (Shanghai, China). The siRNA sequences used were as follows: p53 siRNA (sense: 5′-CCUGUGCAGUUGUGGGUCATT-3′ and antisense: 5′-UGACCCACAACUGCACAGGTT-3′). Negative control siRNA (sense: 5′-UUCUCCGAACGUGUCACGUTT-3′ and antisense: 5′-ACGUGACACGUUCGGAGAATT-3′). The pcDNA3.1(+)-p53 plasmid was cloned via the insertion of the p53 gene sequence (GenBank Accession No: NM_030989.3) into pcDNA3.1(+). In addition, total mRNA from PC-12 cells was used as a template for cDNA and was used to amplify the p53-coding sequence. The p53 specific forward and reverse primers were forward 5′-CGCGGATCCACCATGGAGGATTCACAGTCGG-3′ and reverse 5′-CCGCTCGAGTCAGTCTGAGTCAGGCCCC-3′, respectively. The amplified p53-coding sequence was digested with BamHI and XhoI, and then ligated into BamHI and XhoI digested pcDNA3.1(+). The sequence of pcDNA-p53 was confirmed by Comate BioTech.

### Transfection and analysis

PC-12 cells were seeded onto 6-well plates at a density of 5 × 10^5^ cells per well for 24 h until the confluence reached 60–80%. Lipofectamine 2000™ reagent was used for transfection according to the manufacturer's instructions. For each transfection, 75 pmoL of p53/control siRNA or 2.5 μg of pcDNA3.1(+)-p53/pcDNA3.1(+) was prepared and overlaid onto the cells separately in DMEM medium and incubated for 6 h at 37°C in a CO_2_ incubator. Then, the medium was removed and replaced with 10% FBS/DMEM and after 42 h, the p53 silencing or overexpression efficiency was evaluated by western blot. The control siRNA and pcDNA3.1(+) were used as negative controls (Lu et al., 2017b).

### Immunofluorescence microscopic examination

The PC-12 cells were pre-transfected with p53 siRNA or pcDNA3.1(+)-p53 for 24 h following the treatment with colistin (125 μg/mL) for 12 and 24 h for immunocytochemistry. Briefly, the cells were fixed in 4% paraformaldehyde for 20 min at 37°C, washed with 0.2% TritonX-100 in PBS, and permeabilized with 1% TritonX-100 for 30 min at 37°C. Then, the cells were washed in PBS and incubated in 5% bovine serum albumin (BSA) for 2 h in blocking buffer. Cells were incubated with p53 or LC3 antibodies (1:150 dilution) overnight at 4°C, washed twice with PBS, followed by incubation with FITC-conjugated goat anti-rabbit IgG (1:400 dilution) for 1 h and 2.5 μg/mL DAPI nuclear stain for 20 min at 37°C. The expression of LC3 was examined under a fluorescence microscope (Nikon Eclipse TE 2000U). The percentage of cells positive for LC3 punctate staining was calculated from counting 100 cells for each experimental group (*n* = 3) as explained in our previous study (Lu et al., 2017c).

### Real-time PCR

Real-Time PCR (RT-PCR) was performed to analyze gene expression using a Biosystem 7500 Real-Time PCR System thermocycler and FastStart Universal SYBR Green Master (ROX) from Roche. Total RNA was extracted using TRI reagent, and 1 μg of RNA was reverse transcribed with a Transcriptor First Strand cDNA Synthesis Kit. Oligo nucleotide primers (Table 1) for beclin1, Atg5 and p62 were designed by Sangon Biotech (Shanghai, China), based on the sequences available in NCBI database. Each sample was analyzed in triplicate. The fold change in gene expression was calculated using theΔΔcycle time (Ct) method (Livak and Schmittgen, 2001) after the expression level of β-actin was normalized.

**Table 1:** Sequences of the oligo nucleotide primers for gene transcription analysis by real-time PCR.

**Gene**	**Primer sequence**	**Product length (bp)**
Atg5	Forward 5′-CCCTGAAGACGGAGAGAAGA-3′	152
	Reverse 5′-TGCTGATGTGAAGGAAGTTGTC-3′	
beclin1	Forward 5′-TGGAAATCACTCGTATCTGGAG-3′	119
	Reverse 5′-CCACCTCTTCTTTGAACTGCT-3′	
p62	Forward 5′-CCTATTACCTGGCCTGTGGA-3′	102
	Reverse 5′-GTTCATCCGTTGTGCATGAG-3′	
β-actin	Forward 5′ ACCGCAAATGCTTCTAAACC-3′	192
	Reverse 5′-CCAATCTCGTCTTGTTTTATGC-3′	

### Western blot

Western blot procedure was carried out as mentioned in our earlier study (Lu et al., 2017a). PC-12 cells were pre-treated as above. To prepare the protein lysates, cells were washed twice with cold PBS, and the total protein extracts were prepared in radio-immunoprecipitation assay (RIPA) lysis buffer containing 0.1% Non-idet P-40 and 0.5 mM PMSF, followed by high-speed centrifugation (12,000 × g for 30 min) at 4°C. Then, the supernatant protein content was determined using BCA protein assay kit (Dai et al., 2013). Equivalent amounts of protein were separated in a 10-15% gel by SDS-polyacrylamide gel electrophoresis (SDS-PAGE) run on 120 v for 1 h, and then transferred onto nitrocellulose (NC) membranes run on 120 v for 90 min. After blocking in 5% non-fat dried milk-TBST (25 mM Tris-HCl, 150 mM NaCl, 0.1% Tween-20; pH 7.4) at room temperature for 1 h, the membranes were incubated overnight on a shaker at 4°C with primary antibodies against: p53 (1:400 dilution), Atg5 (1:500 dilution), LC3-II/I (1:1,000 dilution) and β-actin (1:1,000 dilution) at 4°C. The secondary anti-mouse or anti-rabbit IgG peroxidase antibody (1:5,000 dilution) was incubated at room temperature for 1 h, and the blots were developed using enhanced chemiluminescence (ECL) detection (Amersham-Pharmacia Biotech). The level of immunoreactivity was assessed as a peak intensity using an image capture and analysis system (GeneGnome, Syngene, UK). The anti-β-actin antibody was used to control the protein quality and ensure equal loading.

### Electron microscopy

Cells were fixed overnight in 2.5% glutaraldehyde. They were washed with PBS twice and post-fixed in 1% osmium tetroxide at 4°C for 1 h. Next, the cells were dehydrated by ethanol series and 100% acetone, embedded in epoxy resins. The ultrathin sections were stained with uranyl acetate and lead citrate (Zhang et al., 2016), and then observed under a GEM-1200ES transmission electron microscope (JEOL Ltd., Tokyo, Japan).

### Hoechst 33258 staining

Hoechst 33258 staining was used to investigate the changes in nuclear morphology of apoptosis, observed by fluorescence microscopy. The cells were fixed with 4% paraformaldehyde for 10 min at 37°C, washed with PBS and stained with 5 μg/mL Hoechst 33258 for 10 min in the dark. The production of blue fluorescence in condensed and fragmented nuclei was counted as apoptotic cells (Zhu et al., 2011).

### Statistical analysis

Data were obtained from three independent experiments and are presented as mean results ± standard deviation (*SD*). The significance was determined using one-way ANOVA followed by LSD and Dunnett's T3 test. The data were analyzed using the SPSS V17.0 statistical software (SPSS, Chicago, IL). Values < 0.05 (*p* < 0.05) were considered statistically significant.

## Results

### Regulation of colistin induced autophagy by silencing of p53 in PC-12 cells

**Figure 1:**
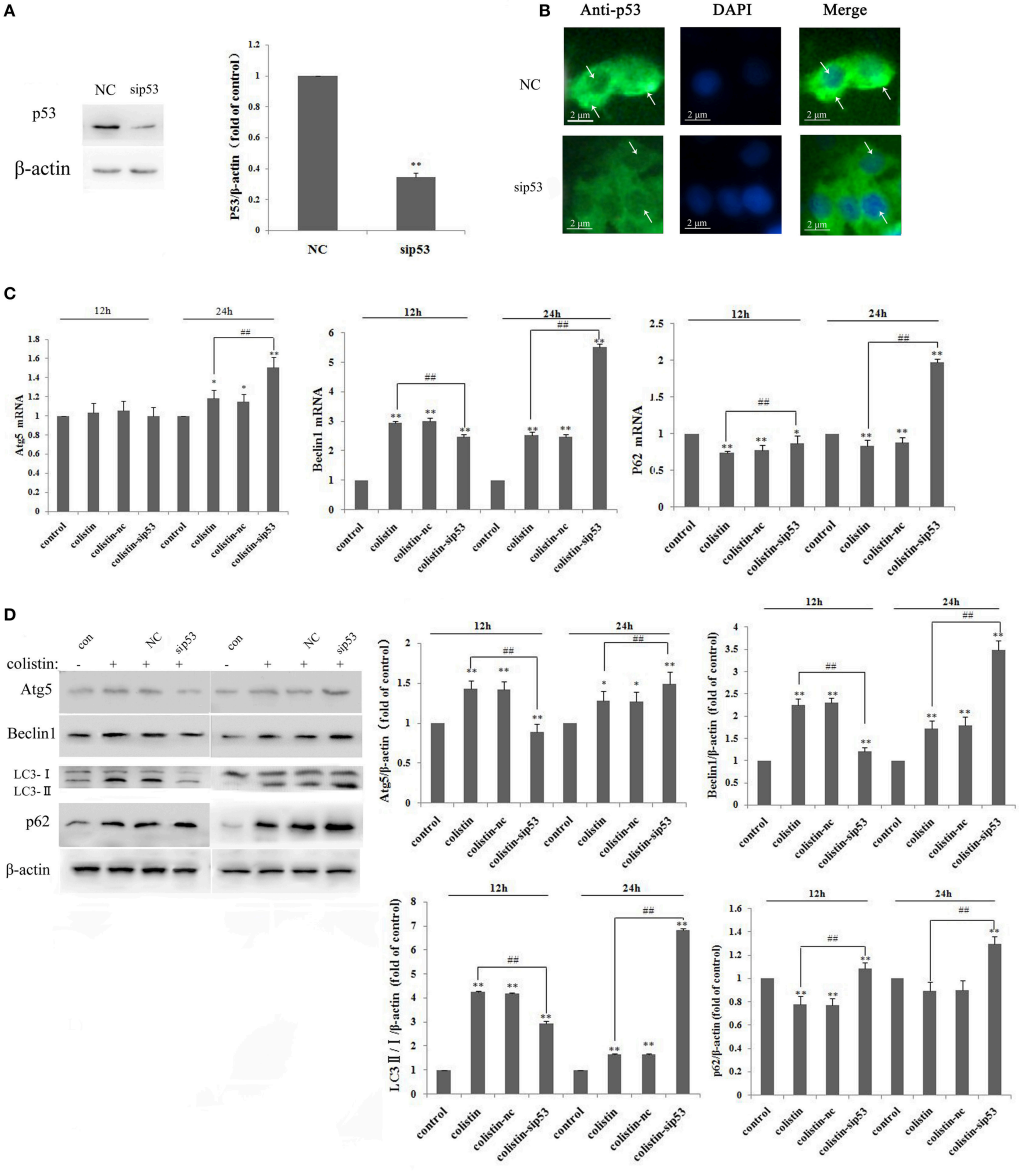
P53-induced impaired autophagy in PC-12-sip53 cells after colistin treatment. **(A)** Western blot assaying on the levels of p53. Quantification of the protein levels following transfection with siRNA-p53, ^**^p < 0.01 vs. control group. Bar graph represents the mean percentage ± *SD* of autophagy cells (*n* = 3). ^**^p < 0.01 vs. control group; ^##^*p* < 0.01 vs. inter-groups. **(B)** The p53 level was decreased in PC-12 cells transfected with sip53 at 48 h, and its localization was determined by immunofluorescence staining with a p53 antibody (green) and DAPI staining (blue). Arrows indicate p53 punctum. **(C)** The expression levels of autophagy-related genes, including Atg5, beclin1, and p62 were determined by RT-PCR following 125 μg/mL colistin treatment for 12 and 24 h. Bar graph represents the mean percentage ± *SD* of autophagy cells (*n* = 3). ^*^p < 0.05 vs. control group, ^**^p < 0.01 vs. inter-groups; ^*^p < 0.05 vs. control group, ^##^*p* < 0.01 vs. inter-groups. **(D)** Western blot assaying on the levels of Atg5, beclin1, p62, and the ratio of LC3-II/I. The β-actin level was used as the internal standard. Bar graph represents the mean percentage ± *SD*, whereas *n* = 3. ^**^p < 0.01 vs. control group.

To elucidate the molecular mechanism of p53 in autophagy, PC-12 cells were transfected with a p53-specific siRNA (sip53) or negative control siRNA (nc), and p53 was effectively silenced (Figure 1A). Figure 1B showed that p53 level in cytoplasm significantly decreased in PC-12 cells after transfection with sip53 according to immunofluorescence staining.

We employed RT-PCR and western blot to determine autophagy-related genes and protein expression levels, respectively. Compared with colistin-treated group, the mRNA expression level of Atg5 and beclin1 were decreased at 12 h but significantly increased at 24 h, whereas p62 mRNA levels were enhanced at 12 and 24 h (Figure 1C; all *p* < 0.01). The western blot results showed that the ratio of LC3-II/I (a marker protein of autophagy) and other autophagy-related proteins significantly increased after colistin treatment for 24 h (Figure 1D), and these results were consistent with RT-PCR analysis.

**Figure 2:**
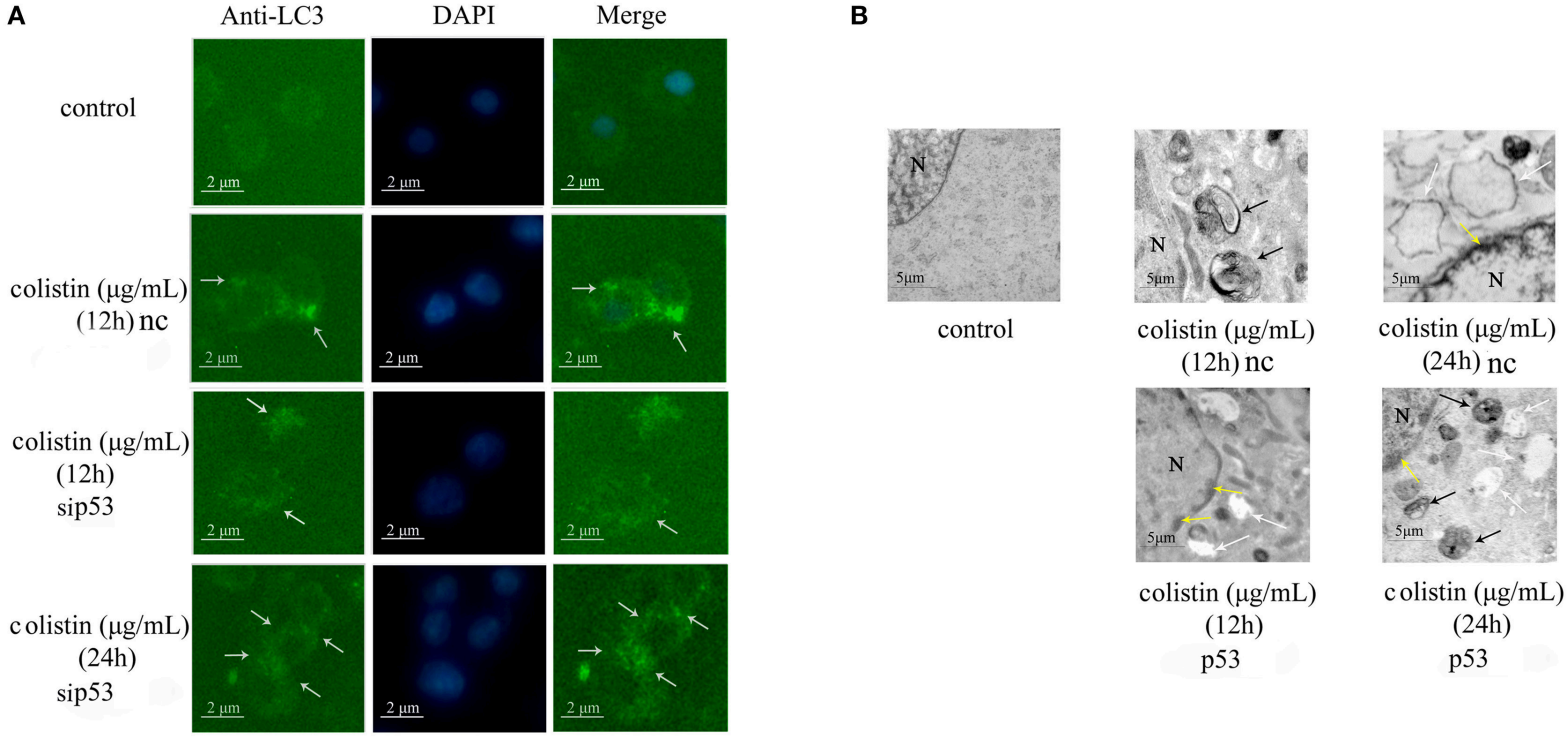
Morphologic analysis of sip53-induced autophagy in colistin-treated PC-12 cells. **(A)** LC3 staining of p53 silenced cells or negative control siRNA following colistin treatment for 12 and 24 h. Arrow indicates LC3 punctum. **(B)** Transmission electron microscopy observations of the control PC-12 cells, PC-12-nc cells treated with colistin at 12 and 24 h and PC-12-sip53 cells treated with colistin (12 and 24 h). Black arrows indicate the typical autophagosomes containing intracellular components and organelles, whereas apoptotic changes such as chromatin condensation (yellow arrows) and cytoplasmic vacuolization (white arrows).

LC3 antibody was used in immunofluorescence microscopy to detect changes of PC-12-sip53 cells. LC3-specific punctate staining (green dots) is indicative of autophagic vacuoles (AVs) formation. Figure 2A depicts immunofluorescence images that show absence of LC3 punctate in the control group. The number of LC3 punctate in PC-12-sip53 cells was less than the colistin-treated PC-12-nc cells at 12 h; whereas different sizes and stages of AVs were substantially increased in colistin-treated PC-12-sip53 cells for 24 h.

Moreover, we used transmission electron microscopy to observe the colistin-induced autophagy in PC-12-nc and PC-sip53 cells. As illustrated in Figure 2B, colistin-untreated PC-12 cells showed normal morphology, with intact nuclear and normal aggregation of organelles. After 12 h of treatment with colistin, there were a large number of autophagic vacuoles of characteristic double or multiple membranes intracellularly (containing cytoplasmic organelles and autophagosomes) in PC-12-nc cells, and nuclear chromatin concentration and cytoplasmic vacuolization were evident after 24 h. Importantly, the number of AVs were diminished in colistin-treated PC-12-sip53 cells at 12 h as compared with colistin-treated PC-12-nc group, but at 24 h had an opposite phenomenon, formation of autophagic aggregates, and marginalization of chromatin has been increased. These data revealed that silencing of p53 cause down-regulation of autophagy in PC-12 cells following colistin treatment for 12 h and up-regulated autophagy for 24 h.

**Figure 3:**
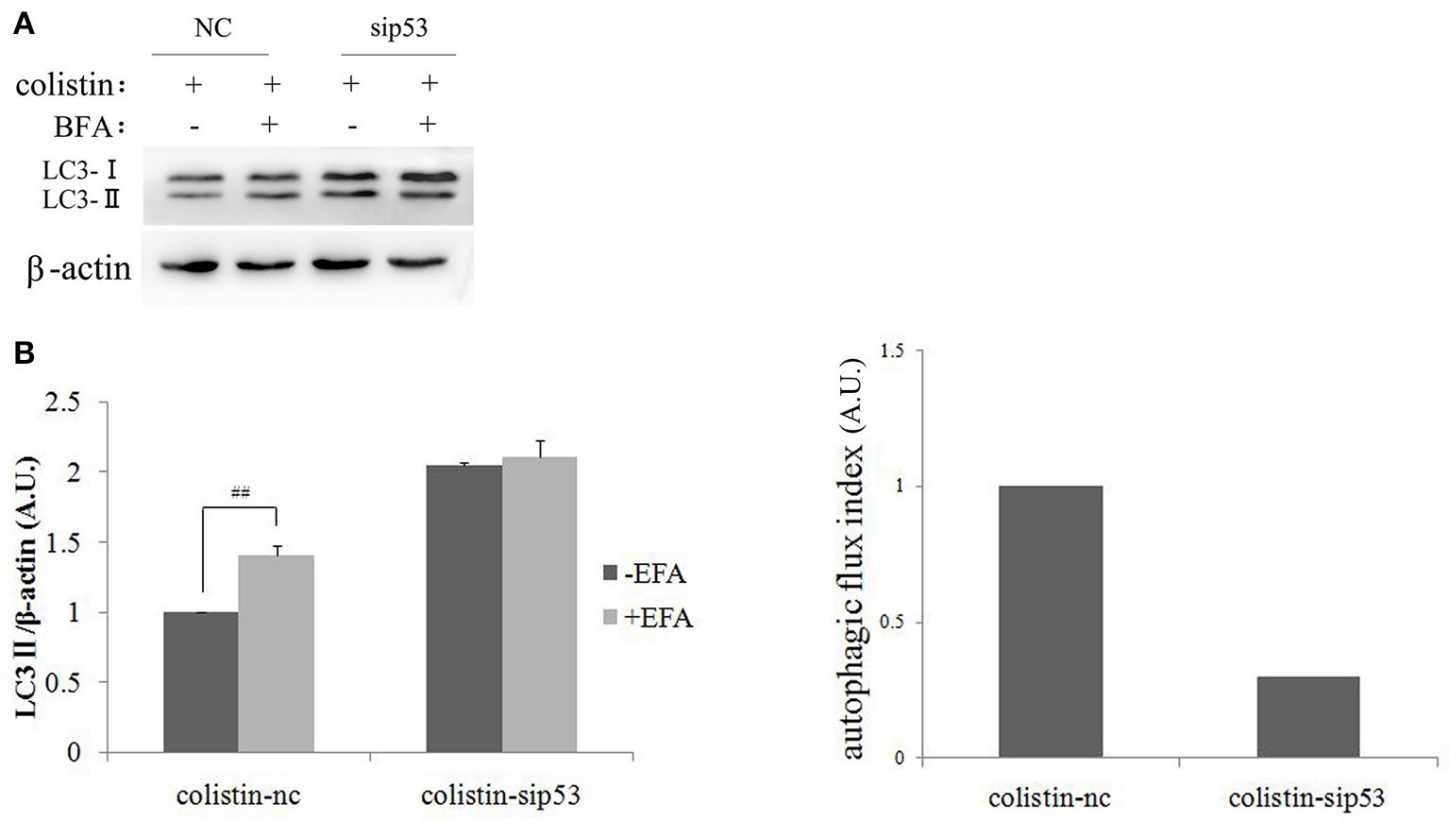
Autophagic flux in PC-12-sip53 cells with colistin treatment. **(A)** PC-12 cells and PC-12-sip53 cells were treated with colistin (125 μg/mL) for 24 h, respectively after the absence and presence of BFA at 3 h. **(B)** Western blot assaying on the levels of LC3-II/I. The β-actin level was used as the internal standard. Bar graph represents the mean percentage ± *SD*, whereas *n* = 3. ^##^*p* < 0.01 vs. control group. **(C)** Autophagic flux indexes were calculated from the data illustrated in **(B)** as the difference in LC3-II/β-actin ratios with or without BFA, expressed in arbitrary units (a.u.).

To further elucidate the exact role of autophagy function after colistin treatment in PC-12-sip53 cells, we assessed autophagy flux. Its indexes were calculated as the difference in the amount of LC3-II with or without BFA (inhibitor of autophagosome and lysosomal fusion) intervention. As shown in Figures 3A,B, PC-12-nc cells treated with colistin after BFA (50 nM in DMSO) had up-regulated the amount of LC3-II to about 1.5-fold as compared to without BFA. Whereas, inhibiting the fusion of lysosomes to autophagosomes in PC-12-sip53 cells exposed to colistin by BFA treatment did not increase the levels of LC3-II. These results suggested that silencing of p53 causes an impairment of autophagy flux in colistin-treated PC-12 cells, and it reduced the autophagic flux index of cells cultures from 1 to about 0.3 (Figure 3C).

**Figure 4:**
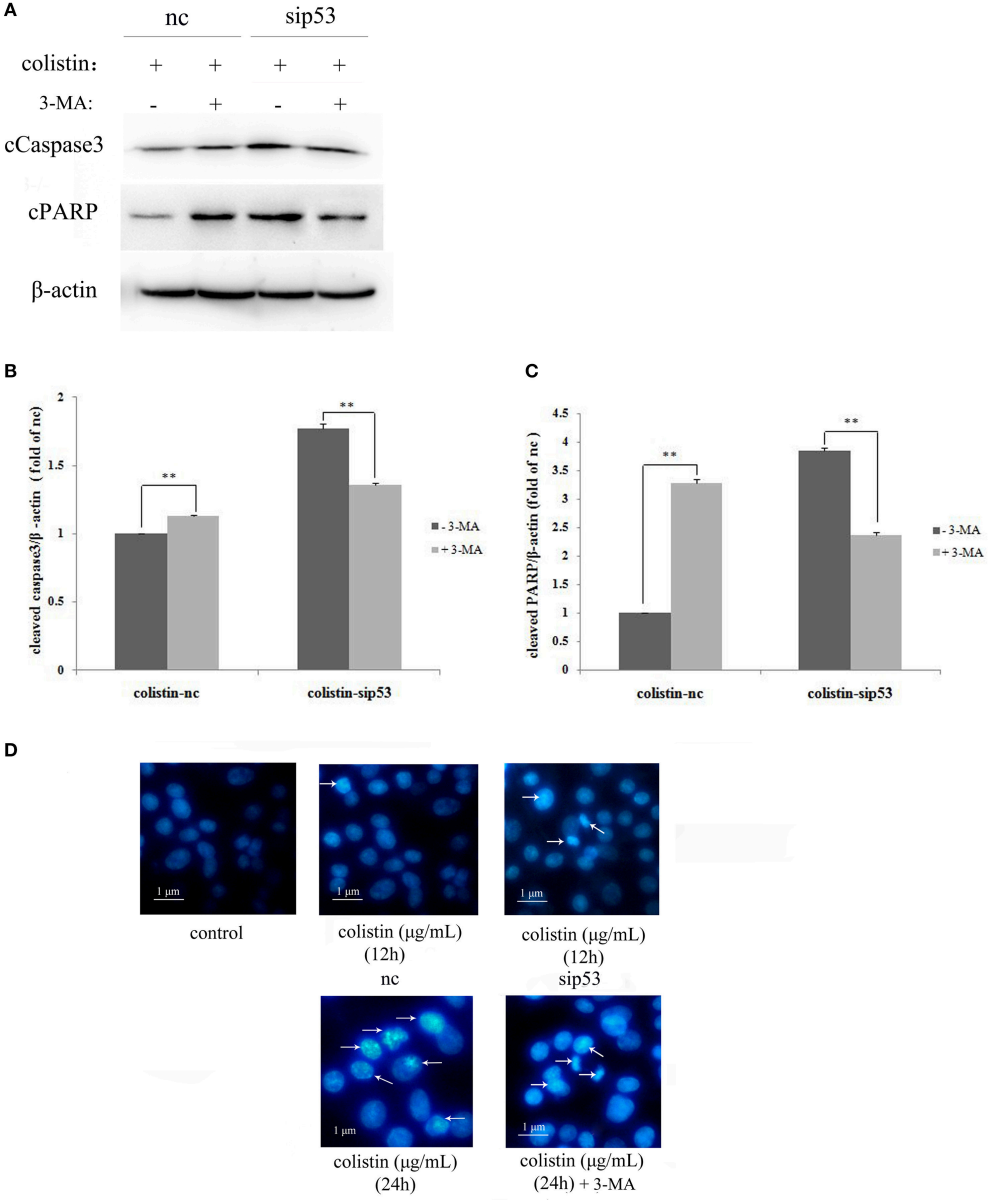
Apoptosis induced by the impaired autophagy in PC-12-sip53 cells treated with colistin. **(A–C)** Lysates of PC-12 cells and PC-12-sip53 cells, untreated or treated 3-MA for 1 h and further treated with colistin (125 μg/mL) at 12 h, and then western blot assaying on the levels of cleaved caspase3 and PARP. The β-actin level was used as the internal standard. Bar graph represents the mean percentage ± *SD*, whereas *n* = 3. ^**^p < 0.01 vs. control group. **(D)** Changes of nuclear morphology in PC-12-sip53 cells with colistin treatment for 12 and 24 h, incubated with or without 3-MA by Hoechst 33258 staining. Arrows indicate fragmentation of the nuclear or karyopyknosis.

To determine whether sip53-induced autophagy affect apoptosis in colistin-treated PC-12 cells, autophagy inhibitior 3-MA (5 mM) was used in PC-12-sip53 cells after colistin treatment for 24 h. As shown in Figures 4A–C the silencing of p53 with colistin treatment for 24 h led to significantly decreased cleavage of caspase3 and PARP in the presence of 3-MA. The data suggested that sip53-induced autophagy could activate apoptosis in PC-12 cells with colistin treatment.

The result of Hoechst 33258 staining shown in Figure 4D, colistin-treated PC-12-nc cells displayed indication of apoptosis; more nuclear fragmentation and chromatin condensation in colistin-treated PC-12-sip53 cells as compared to the control cells, in particular at both time points (12 h and 24 h), and showed peak results at 24 h (Figure 4D), but 3-MA pre-treatment exposure reduced chromatin condensation at 24 h. As expected, sip53-induced autophagy was blocked by 3-MA results in down-regulation of apoptosis in colistin-treated PC-12 cells.

### Regulation of colistin-induced autophagy by overexpression of p53 protein in PC-12 cells

**Figure 5:**
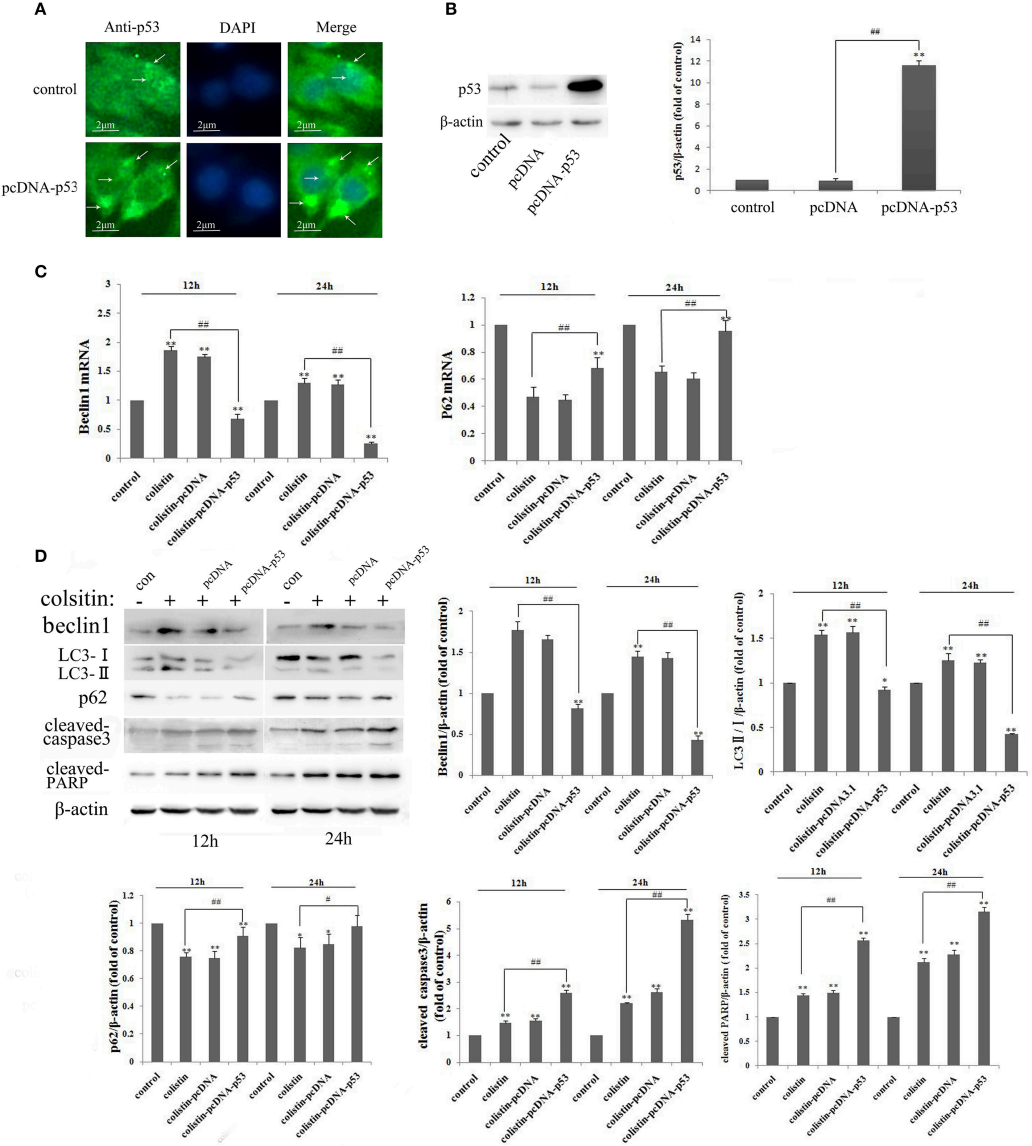
Autophagy and apoptosis in PC-12-p53 cells after colistin treatment. **(A)** The expression levels of p53 were increased after transfection with a p53 recombinant plasmid, and p53 was localized by immunofluorescence staining with a p53 antibody (green) and nuclear staining with DAPI (blue). Arrows indicate p53 punctum. **(B)** The expression levels of p53 were assayed by western blot and quantification of the p53 protein levels. ^**^p < 0.01 vs. control group; ^##^*p* < 0.01 vs. inter-groups. **(C)** The expression levels of autophagy-related genes, including beclin1 and p62, were determined by RT-PCR following colistin treatment for 12 and 24 h. Bar graph represents the mean percentage ± *SD* of autophagy cells (*n* = 3). ^**^p < 0.01 vs. control group; ^##^*p* < 0.01 vs. inter-groups. **(D)** The expression levels of beclin1, p62, the ratio of LC3-II/I and cleaved caspase3 and PARP by western blot. The β-actin level was used as the internal standard, and shows the quantitative results of the expression levels, respectively. ^*^p < 0.05; ^**^p < 0.01 vs. control group; ^#^*p* < 0.05; ^##^*p* < 0.01 vs. colistin alone group.

We used the manipulated genetic approach to gain further insight into the effect of p53 in colistin-induced neurotoxicity. As shown in Figure 5A, overexpression of p53 was mainly localized in the cytoplasm based on immunofluorescence staining. Quantitative densitometry of the western blot for p53 revealed that it was significantly up-regulated in the p53 overexpressing (PC-12-p53) group (Figure 5B, *p* < 0.01). It is noted that expression levels of the positive regulators of autophagy (LC3-II/I and beclin1) were reduced after colistin treatment in PC-12-p53 cells in a time-dependent manner, compared to the colistin-treated group; the p62 expression levels were significantly increased from 12 to 24 h (Figures 5C,D all *p* < 0.01). As shown in Figure 5D, cleavage of the pro-apoptotic proteins caspase3 and PARP was pronounced after colistin treatment for 12 and 24 h in PC-12-p53 cells, and showed the highest level at 24 h as compared to the colistin-treated group (*p* < 0.01). These data indicate that overexpression of p53 inhibits colistin-induced autophagy and has a promoting apoptotic effect on colistin-treated cells.

**Figure 6:**
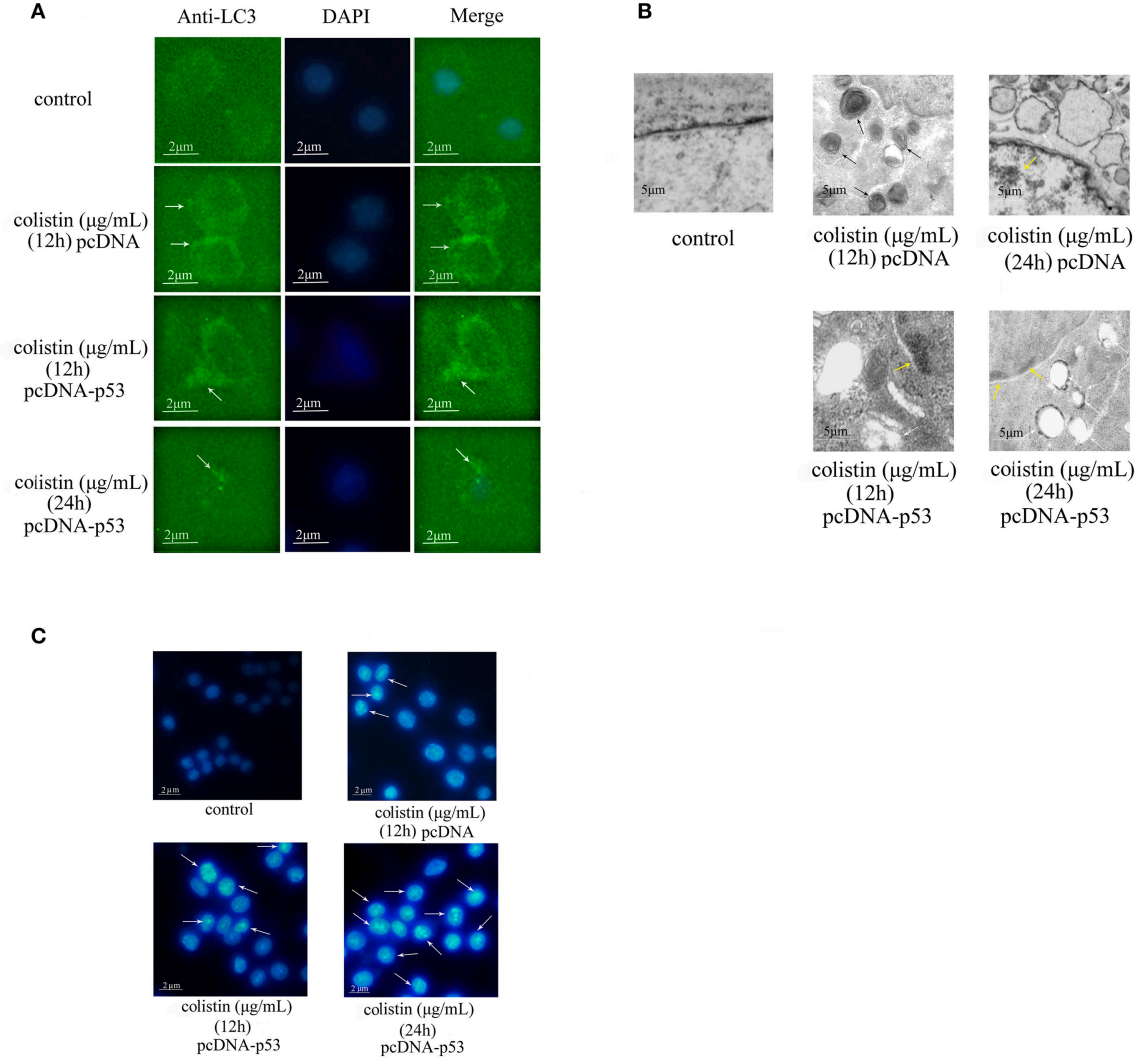
Morphologic analysis of p53-induced autophagy in colistin-treated PC-12-p53 cells. **(A)** LC3 staining of p53 overexpressing cells following 125 μg/mL colistin treatment for 12 and 24 h. The control group was transfected with null vector. Arrow indicates LC3 punctum. **(B)** Transmission electron microscopy observations of the control PC-12 cells, PC-12-pcDNA cells treated with colistin at 12 and 24 h and PC-12-p53 cells treated with colistion (12 and 24 h). Autophagosomes, cytoplasmic vacuolization, and chromatin condensation in the treated groups are marked with black, white, and yellow arrows respectively. **(C)** Hoechst 33258 staining showed changes in the nuclear morphology of cells treated with colistin for 12 and 24 h. Arrows indicate fragmentation of the nuclear or karyopyknosis.

In addition to fluorescence microscopy, electron microscopy was employed to further observe colistin-induced autophagy in PC-12-pcDNA and PC-12-p53 cells. LC3 dots displayed as shown in Figure 6A, the autophagy biomarker LC3-II was reduced significantly by overexpression of p53 in colistin-treated cells as compared to colistin-treated PC-12-pcDNA cells. Similarly, the images of electron microscopy shown in Figure 6B, overexpression of p53 in colistin treatment at 12 h led to few autophagic vacuoles, the further 12 h overexpression of p53 resulted in autophagosomes not obvious, and nuclear chromatin condensation and cytoplasmic vacuolization evident in colistin-treated PC-12-p53 cells.

Furthermore, the Hoechst 33258 staining results showed that apoptosis gradually appeared and highly enhanced at 24 h as compared to the colistin-treated group (Figure 6C). These results lend further support to the verdict that overexpression of p53 inhibits colistin-induced autophagy and that it could accelerate apoptosis in PC-12 cells.

## Discussion

The ability of autophagic system to recycle nutrients, maintain cellular energy homeostasis, and degrade toxic cytoplasmic constituents (and bacterial infection) helps to keep cells alive during nutrient and growth factor deprivation and other stressful conditions (Levine and Klionsky, 2004). Therefore, understanding the complex functionality of autophagy induction is highly important. In our previous study, we proved that colistin (125 μg/mL) induces a high level of autophagy at 12 h and that autophagy can protect against the colistin-induced apoptosis (Jiang et al., 2014), and Zhang et al. reported that colistin increased the expression level of p53, and p53 involved in colistin-induced autophagy and apoptosis (Zhang et al., 2016). In this follow-up mechanistic study, we are interested in exploring whether p53-mediated autophagy plays a cell survival role in colistin-treated PC-12 cells and p53 could act as a target gene to protect against colistin-induced neurotoxicity.

p53, a tumor suppressor protein, plays a critical role in cellular response to acute stress. In the physiological condition, Mdm2-mediated ubiquitination and proteasomal degradation control p53 levels (Ryan, 2011). It rapidly undergoes reversible post-translational modifications for its stabilization in response to hypoxic, genotoxic, and oncogenic stress (Kruse and Gu, 2009). Active p53 in the nucleus binds to the activator region of (and hence transactivate) plethora target genes involved in progression of cell cycle, apoptosis, and metabolism (Vousden, 2007). Additionally, p53 mediates transcription-independent extra nuclear onco suppressing functions (Green and Kroemer, 2009). In addition, p53 has a bidirectional modulatory action on autophagy, it has been demonstrated that p53-induced autophagy may lead to diametrically opposite results, such as physiological cellular defense response (Kerr et al., 1972) or causing cell death (Wang et al., 2011), depending on the p53 subcellular localization (Tang et al., 2015). In the present study, we used siRNA against p53 mRNA sequence to silence p53 in PC-12 cells, and overexpressed p53 through a recombinant plasmid. Immunofluorescence staining was employed to observe the localization of p53, owing to the localization of p53 in cells directly related with its role in the regulation of autophagy or apoptosis. Especially, we found that siRNA/recombinant-plasmid transfected into PC-12 cells was localized in the cytoplasm rather than in the nucleus. These results provide basis for future research to study the role of p53 in colistin-induced autophagy in PC12 cells.

It has been stated that cytoplasmic p53 acts as an inhibitor of autophagy via protein-protein interactions in mitochondria (Green and Kroemer, 2009), but acts as an activator of autophagy by transactivating its target genes after majority of p53 translocates to the nucleus in response to various forms of cellular stresses (Crighton and Ryan, 2004; Sui et al., 2011). P53 inhibits the negative regulator of autophagy mTOR (mammalian target of rapamycin) via AMP-activated protein kinase (AMPK) pathway activation, which in turn induces autophagy. Damage-regulated autophagy modulator (DRAM) acts as a downstream gene of p53 in the control of autophagy and cell death. Besides the discovery of death-associated protein kinase 1 (DAPK-1) can favor autophagy induction by binding to LC3 (Harrison et al., 2008), the BH3 domain of beclin1 is phosphorylated by DAPK-1 in the release of beclin1 from Bcl2/Bcl-xl, thereby stimulating autophagy (Zalckvar et al., 2009a,b). Notably, it is determined that p53 is a pro-autophagic factor that promotes autophagy via transactivating its target genes, but the pre-conditions are various cellular stresses to activate p53 and translocate it from cytoplasm into the nucleus (Green and Kroemer, 2009). However, autophagy inhibition by p53 is more obvious when the nuclear localization sequence of p53 is deleted, leading to a merely cytoplasmic p53 localization (Ryan, 2011). In our present study, it is clear from the results that autophagy-related genes and protein were inhibited in colistin-treated PC-12-sip53 cells at 12 h, but opposite results occurred at 24 h, indicating that p53 down-regulated autophagy in PC-12-sip53 cells at 12 h and up-regulated autophagy at 24 h. Our previous study showed that colistin induced the highest expression levels of nuclear p53 after 12 h, but it induced the highest expression of cytoplasmic p53 at 24 h (Zhang et al., 2016). Thus, we inferred that the neurotoxicity of colistin acted as a stress/stimulation in PC-12 cells to be a signal of p53 translocation into the nucleus, as p53 expression levels was reduced by siRNA, leading to the lower levels of p53-translocated into nucleus, thereby silencing of p53 down-regulated colistin-induced autophagy in PC-12 cells for 12 h. Thereafter, cytoplasmic p53 plays a leading role in down-regulation of autophagy, while the relatively decreased cytoplasmic p53 level resulted in activation of autophagy in PC-12-sip53 cells at 24 h.

The term “autophagic flux” describe that autophagy is a dynamic process (Zhou et al., 2017), and there is a dynamic equilibrium between autophagosome formation and clearance by lysosome. Actually, the rate at which material is cleared from the cell by autophagy is known as autophagy flux (Palumbo et al., 2016). The stability of LC3-II levels or amount of AVs is the net effect of both AV maturation and degradation (Komatsu et al., 2007). Therefore, the turnover of LC3 in autophagy has been widely used to monitor autophagic flux. In addition, there is an inverse relationship between p62/SQSTM1 levels and autophagy, because it is localized at the autophagic compartments and is degraded by autophagy-lysosomal pathway, and the accumulation of p62 accompanies the impairment of autophagy in cells (Komatsu et al., 2007; Mizushima and Yoshimori, 2007; Nakai et al., 2007; Mizushima et al., 2010). In current study, silencing of p53 up-regulated colistin-induced autophagy at 24 h (including the accumulation of AVs and significantly increased autophagy related genes), but the p62 level was dramatically increased, suggesting the blockage of lysosomal degradation. Therefore, we inferred that autophagy may be impaired. Indeed, our result showed an increase of colistin-induced LC3-II accumulation in PC-12 cells following the addition of the late-stage autophagy inhibitor (BFA). In contrast, the index of autophagic flux (be quantified by measuring LC3 turnover) is barely increased (Figure 3C), which indicated the impairment of sip53-induced autophagy in colistin-treated PC-12 cells. Our results are in line with the study conducted by Mizushima et al. that a concurrent increase has been noted in these markers, and being consistent with an impairment of autophagosome clearance (Mizushima et al., 2010), which is blocked in the last stage of autophagy. Furthermore, we found that colistin alone group had a significant elevating effect on autophagy that caused cell death in PC-12-sip53 cells, but pretreatment of the cells with 3-MA decreased apoptosis, suggesting a pro-apoptotic role of autophagy, as measured by cleavage of caspase3 and PARP, 33258 staining (Figures 4A–D). These results suggested that apoptosis is able to be activated by silencing of p53 up-regulation autophagy, ultimately, leading to cell death rather than PC-12 cells survival. Furthermore, beclin1 is autophagy-related protein 6 and is a key factor in the initial formation of autophagosomes (Huang et al., 2016). However, another study demonstrated that beclin1 is a substrate of caspase3 and cleaved beclin1 begins to promote apoptosis (Li et al., 2016), which may be also a cause of autophagic cell death in colistin-treated PC-12-sip53 cells.

In the same manner, immunocytochemistry analysis showed that overexpression of p53 is only localized to the cytoplasm because the recombinant plasmid may be not integrated into the genome of PC-12 cells (Figure 5A). In a recent study, it has been reported that cytoplasmic p53 is an inhibitor of autophagy in cells, and it inhibits basal autophagy dependent protein-protein interactions in the mitochondria rather than transactivate its target genes (Tasdemir et al., 2008a,b). Indeed, in our study, down-regulation of the autophagy markers (including LC3 and beclin1) and up-regulating the cleavage of caspase3 and PARP, and 33258 staining revealed that the increased expression levels of cytoplasmic p53 inhibited colistin-induced autophagy in PC-12 cells and induced apoptosis (Figures 5C, D, 6A–C). A number of studies had confirmed that ROS generation, mitochondrial dysfunction and release of cytochrome *c* were triggered by the neurotoxicity of colistin, which was the cause of apoptosis (Livak and Schmittgen, 2001; Jiang et al., 2014). The most cytoplasmic p53 translocate to mitochondria leading to the up-regulated expression of caspase3 and ratio of Bax/Bcl2 following colistin treatment, and thus accelerates apoptosis (Jiang et al., 2014). Meanwhile, colistin-induced neurotoxicity has a positive role in up-regulating the expression of p53, which eventually further speed up apoptosis. Thus, overexpression of cytoplasmic p53 up-regulation colistin-induced apoptosis maybe associated with p53-dependent mitochondrial apoptotic pathway in PC-12 cells.

## Conclusion

This is the first study which demonstrated the silencing of p53 inhibits autophagy in PC-12 cells at 12 h and then up-regulates defective autophagy from 12 to 24 h, and eventually promotes apoptosis. Overexpression of p53 inhibits colistin-induced autophagy, and accelerates apoptosis in a time-dependent manner in PC-12 cells. Hence, we suggest that p53 plays a negative role in regulating colistin-induced autophagy in PC-12 cells and it could not act as a neuro-protective target in colistin-induced neurotoxicity.

## Author contributions

JL supervised the whole experiments. ZL, CC, and ZW designed this study and contributed to the paper writing. YM, LD, ET, WH, HN, RL, and BW performed the practical work and completed the experiments. IM helped in revising and improving the language expression.

### Conflict of interest statement

The authors declare that the research was conducted in the absence of any commercial or financial relationships that could be construed as a potential conflict of interest.
